# Inflammatory mechanisms and therapeutic advances in chronic endometritis

**DOI:** 10.3389/fimmu.2025.1616217

**Published:** 2025-09-05

**Authors:** Xinyang Yan, Jiao Jiao, Xiuxia Wang

**Affiliations:** ^1^ Center of Reproductive Medicine, Shengjing Hospital of China Medical University, Shenyang, China; ^2^ Shenyang Reproductive Health Clinical Medicine Research Center, Shenyang, China

**Keywords:** chronic endometritis, endometrial microbiome, TLR/NF-κB pathway, NLRP3 inflammasome, DNA methylation, antibiotic resistance

## Abstract

Chronic endometritis (CE) is a persistent inflammatory disorder of the endometrium, associated with infertility, recurrent pregnancy loss, and implantation failure. Diagnosis primarily depends on hysteroscopy and immunohistochemistry, while microbial dysbiosis and antibiotic resistance pose significant challenges to effective management. The pathogenesis of CE involves microbial infections that induce immune dysregulation through TLR/NLR signaling pathways, metabolic reprogramming of immune cells, miRNA-mediated inflammatory responses, and DNA methylation alterations. The activation of pro-inflammatory mediators and the NLRP3 inflammasome further aggravates endometrial dysfunction. Treatment typically includes oral antibiotics and intrauterine therapies, although their efficacy is variable. Probiotics have demonstrated potential in restoring microbial balance. This review outlines the inflammatory mechanisms underlying CE and recent therapeutic advancements, highlighting potential targets for improving treatment outcomes.

## 1 Introduction

Chronic endometritis (CE) is a persistent inflammatory condition localized to the endometrium, strongly linked to adverse pregnancy outcomes, including infertility, recurrent pregnancy loss, and recurrent implantation failure (1, 2). Diagnosis primarily relies on hysteroscopic examination and immunohistochemical staining. Common hysteroscopic and histological manifestations of CE include stromal edema, focal congestion, increased stromal cell density, and infiltration of abnormal plasma cells in the endometrial stroma (3, 4).

Recent developments have shifted the understanding of CE from a purely infectious etiology to a complex immunological disorder (5). Microbial dysbiosis within the endometrium disrupts microbial balance and triggers dysregulated immune responses involving both innate and adaptive immunity (6, 7). Key inflammatory pathways, such as Toll-like receptors (TLRs), NOD-like receptors (NLRs), and downstream NF-κB signaling, alongside inflammasome activation and metabolic reprogramming of immune cells, are central to the persistence of chronic inflammation and impaired endometrial receptivity (8–10). Therapeutically, empirical antibiotic regimens, such as doxycycline and metronidazole, have shown efficacy in histological resolution and partial improvement in reproductive outcomes (11, 12). Several studies report significant increases in clinical pregnancy and live birth rates following antibiotic treatment in women with CE undergoing *in vitro* fertilization (IVF) (13). However, other studies indicate that a subset of patients with CE experience persistent inflammation or poor reproductive outcomes despite standard treatment (14, 15), highlighting the heterogeneity of treatment responses and underscoring the need for adjunctive strategies such as intrauterine therapy or immunomodulation.

Despite advancements in characterizing CE pathophysiology and developing management strategies, several knowledge gaps persist. The absence of standardized diagnostic criteria, variability in therapeutic response, and limited understanding of immune-microbiota interactions continue to impede effective clinical translation. This review synthesizes current insights into the immunological mechanisms underlying CE, with a focus on TLR/NLR signaling, immune cell metabolic rewiring, miRNA-mediated inflammation, and epigenetic dysregulation, while evaluating recent therapeutic advances, including antibiotics, intrauterine infusion, and probiotic-based approaches. By summarizing mechanistic and clinical evidence, this review provides a comprehensive framework for guiding future diagnostic and therapeutic innovations in CE.

## 2 Causes of chronic endometritis

### 2.1 Microbial infection

CE is characterized by a localized active infection in the endometrium, disrupting the balance between the uterine microbiome and immune system. Traditional perspectives have emphasized the cervix as a key barrier between the uterus and vagina, with the dominance of lactobacilli in the vaginal microbiota maintaining uterine sterility by suppressing pathogenic microorganisms. Cicinelli et al. (16) used microbial culture techniques to detect a variety of microorganisms in the endometrium of patients with CE, including *Streptococcus*, *Enterococcus faecalis*, *Escherichia coli*, and *Ureaplasma urealyticum*, thus confirming the presence of microbial communities within the uterine cavity. Common pathogens associated with acute endometritis, such as *Chlamydia trachomatis* and *Neisseria gonorrhoeae*, are typically introduced into the uterine cavity through ascension from the vaginal microbiota (17). However, these pathogens are rarely detected in patients with CE, suggesting that the pathogenesis of CE differs from that of acute endometritis. The presence of microorganisms within the uterine cavity is now widely accepted, and given the effectiveness of antibiotic therapy, microbial infection is considered a primary contributor to CE. However, in some cases, endometrial pathogen cultures are negative, and antibiotic treatments fail, implying that multidrug-resistant organisms may play a role in the development of CE (18).

### 2.2 Non-infectious factors

Recent research also highlights that non-infectious factors, including immune dysfunction, endocrine disorders, and environmental influences, may contribute to CE pathogenesis (19–21). Elevated levels of pro-inflammatory cytokines and an increased presence of immune cells, such as T-helper 17 (Th17) cells and M1 macrophages, are commonly observed in CE, indicating that immune system dysfunction may perpetuate the condition (22, 23). Hormonal imbalances, such as those seen in endometriosis and elevated estrogen levels, may influence the susceptibility and severity of CE (24). These endocrine disruptions can affect immune cell function, endometrial receptivity, and inflammatory responses, further contributing to the chronicity of the disease (19). Additionally, exposure to environmental factors, such as smoking (25), may impact immune function and the microbiome (26), potentially exacerbating CE. The interaction between environmental toxins and the immune system may contribute to altered immune responses, increasing the endometrium’s susceptibility to chronic inflammation.

## 3 Immune cells and cytokines in the pathogenesis of CE

### 3.1 TLR and NLR in the pathogenesis of chronic endometritis

Lipopolysaccharide (LPS) is a key pathogen-associated molecular pattern (PAMP) involved in the pathogenesis of CE. Elevated expression of pro-inflammatory cytokines and chemokines has been observed in both tissues and LPS-stimulated endometrial cells of patients with CE (27). Transcriptomic analyses further reveal the enrichment of inflammation-related gene sets, particularly those involved in TLR and NLR signaling (8). LPS activates pattern recognition receptors (PRRs), triggering the MyD88/NF-κB and TRIF/IRF pathways, resulting in sustained production of IL-6, TNF-α, and CXCL8. This signaling cascade creates a chronic pro-inflammatory microenvironment characterized by cytokine accumulation, immune cell infiltration, and disrupted epithelial-stromal interactions (28–30).

#### 3.1.1 Abnormal activation of TLR pathways in chronic endometritis

TLRs play a critical role in pathogen recognition, with TLR4 specifically binding LPS and TLR2 detecting a broader range of microbial PAMPs. Both receptors are mechanistically implicated in CE pathogenesis (31). Endogenous damage-associated molecular patterns (DAMPs), such as HMGB1 and heat shock proteins released from necrotic cells, bind to TLR2, TLR4, or their heterodimeric complexes (32, 33). Moreover, HMGB1-pathogen/DNA complexes interact with advanced glycation end-product (AGE) receptors on antigen-presenting cells, activating TLR7/TLR9 signaling cascades (34). This molecular interaction suggests that microbial infections may trigger the release of modified host-derived molecules that perpetuate inflammatory responses through sustained activation of TLRs and other PRRs, even after pathogen clearance. Such mechanisms may contribute to secondary autoimmune reactions, maintaining chronic inflammation in CE. Pathological overactivation of TLR signaling pathways has been shown to accelerate CE progression (35). Upon PAMP recognition, TLRs initiate downstream signaling through both MyD88-dependent and independent pathways, resulting in NF-κB and MAPK activation (36, 37). These transcriptional regulators subsequently upregulate pro-inflammatory cytokine production, sustaining leukocyte infiltration.

*In vitro* studies provide evidence for the central role of NF-κB in the pathogenesis of endometrial inflammation (9, 10). Pharmacological studies demonstrate that Epimedium glycosides alleviate LPS-induced endometritis by dual modulation of TLR4/NF-κB inhibition and Nrf2 activation (38). Furthermore, dysregulation of TLR signaling components, such as Akt1 deficiency, enhances MyD88 phosphorylation, potentiating NF-κB and interferon regulatory factor activity and amplifying inflammatory cytokine production (39). This highlights the pivotal role of TLR signaling in CE persistence. Aberrant TLR activation not only initiates inflammation but also perpetuates an imbalanced immune response, reinforcing the chronic nature of CE.

#### 3.1.2 NLR pathway dysregulation in chronic endometritis

NLRs, expressed in both immune and non-immune cells, detect cytoplasmic PAMPs and biomolecules. NLRP1 and NLRP2 recognize bacterial cell wall degradation products, while NLRP3 forms inflammasomes in response to a range of stimuli, activating caspase-1 to promote the release of IL-1β and its precursor. NLRP3-driven inflammation contributes to reproductive pathologies, including endometriosis, polycystic ovary syndrome (PCOS), and RPL. NLRP3 activation has been identified in fibrotic ovarian tissues of PCOS mice and in the endometrial tissues of patients with idiopathic RPL (40), suggesting its involvement in chronic inflammation. *In vitro* studies of LPS-stimulated bovine endometrial epithelial cells (BEECs), stromal cells, and peripheral blood mononuclear cells (PBMCs) show increased IL-1β secretion, particularly in stromal fibroblasts (41). Inhibition of NLRP3 or caspase-4 through siRNA blocked IL-1β production. A murine CE model confirmed that LPS-induced endoplasmic reticulum (ER) stress activates TXNIP, which in turn triggers NLRP3 and IL-1β expression (42). LPS-exposed goat endometrial stromal cells exhibited upregulated ER stress, autophagy, and inflammatory markers, effects reversible by the ER stress inhibitor 4-phenylbutyrate (43). However, direct evidence of NLRP3 inflammasome activation in human CE endometrial tissue remains absent, with most findings extrapolated from animal models or *in vitro* studies, limiting their clinical applicability. This lack of clinical evidence constitutes a significant barrier to fully understanding NLRP3’s role in human CE pathogenesis. Addressing this gap through rigorous studies of human endometrial specimens is crucial for validating these pathways and guiding targeted therapeutic approaches.

The regulation of NLRP3 involves multiple mechanisms. ER stress (43), oxidative stress, and inflammation upregulate NLRP3 and pro-IL-1β through TLR pathways, with NLRP3 inflammasome activation occurring once a threshold is reached (44, 45). Co-incubation of HMGB1 with trophoblasts increases NLRP3 expression, indicating that NLR pathway activation drives inflammation (46–48). Elevated extracellular ATP in epithelial cells also activates NLRP3 in uterine macrophages, linking it to sterile inflammation (49, 50). While the precise role of NLRP3 in the initiation of CE remains unclear, it may modulate the Th17/Treg balance, as observed in patients with RPL (51) and CE (52), potentially altering the immune environment of the endometrium. These findings highlight the critical role of NLR pathways, particularly NLRP3 activation, in amplifying innate immune signaling and inflammatory cascades in CE.

### 3.2 Metabolic alterations in endometrial immune cells

Previous studies suggest significant alterations in immune cell subsets in CE, with notable increases in pro-inflammatory cells such as effector T cells and M1 macrophages (53, 54). Immune cell phenotype stability and function are closely linked to metabolic states, highlighting specific metabolic reprogramming events as central drivers of endometrial immune imbalance. This includes enhanced glycolytic flux in effector T cells and M1 macrophages, coupled with reduced fatty acid oxidation in Tregs and M2 macrophages. These shifts promote a pro-inflammatory environment characterized by Th1/Th17 cell dominance (55), diminished Treg suppressive function, and increased reactive oxygen species production. These changes collectively sustain a chronic inflammatory microenvironment, marked by altered cytokine/chemokine profiles and a disrupted immune cell spatial distribution in CE tissues. Increased glycolysis supports the proliferation and migration of pro-inflammatory effector T cells and M1 macrophages, while inhibiting FOXP3 expression and Treg stability (56), further exacerbating inflammation. PAMPs activate TLRs and T cell receptors, modulating mTOR signaling in macrophages (57). In contrast, TGF-β and IL-4 suppress glycolysis, promoting mitochondrial and fatty acid oxidation to sustain anti-inflammatory Tregs and M2 macrophages (58). In CE, reduced levels of TGF-β/IL-4 may amplify glycolysis, further driving inflammation. Additionally, lipid biosynthesis influences immune responses, as LPS-induced activation of SREBP1 reprograms macrophage lipid metabolism, resolving inflammation through unsaturated fatty acid biosynthesis while suppressing TR4/NF-κB pathway genes (59, 60). However, the role of lipid metabolic changes in CE immune cells and their pathogenic contribution remains unclear. Excessive activation of inflammatory pathways in CE likely modulates immune cell proliferation, differentiation, and function through metabolic shifts, perpetuating immune dysregulation and endometrial inflammation. In summary, immune-metabolic reprogramming sustains CE by promoting pro-inflammatory phenotypes and weakening anti-inflammatory resilience, effectively bridging microbial sensing with persistent immune dysfunction.

### 3.3 MicroRNA-mediated inflammation development in chronic endometritis

#### 3.3.1 miRNAs regulating inflammatory pathways

**Figure 1:**
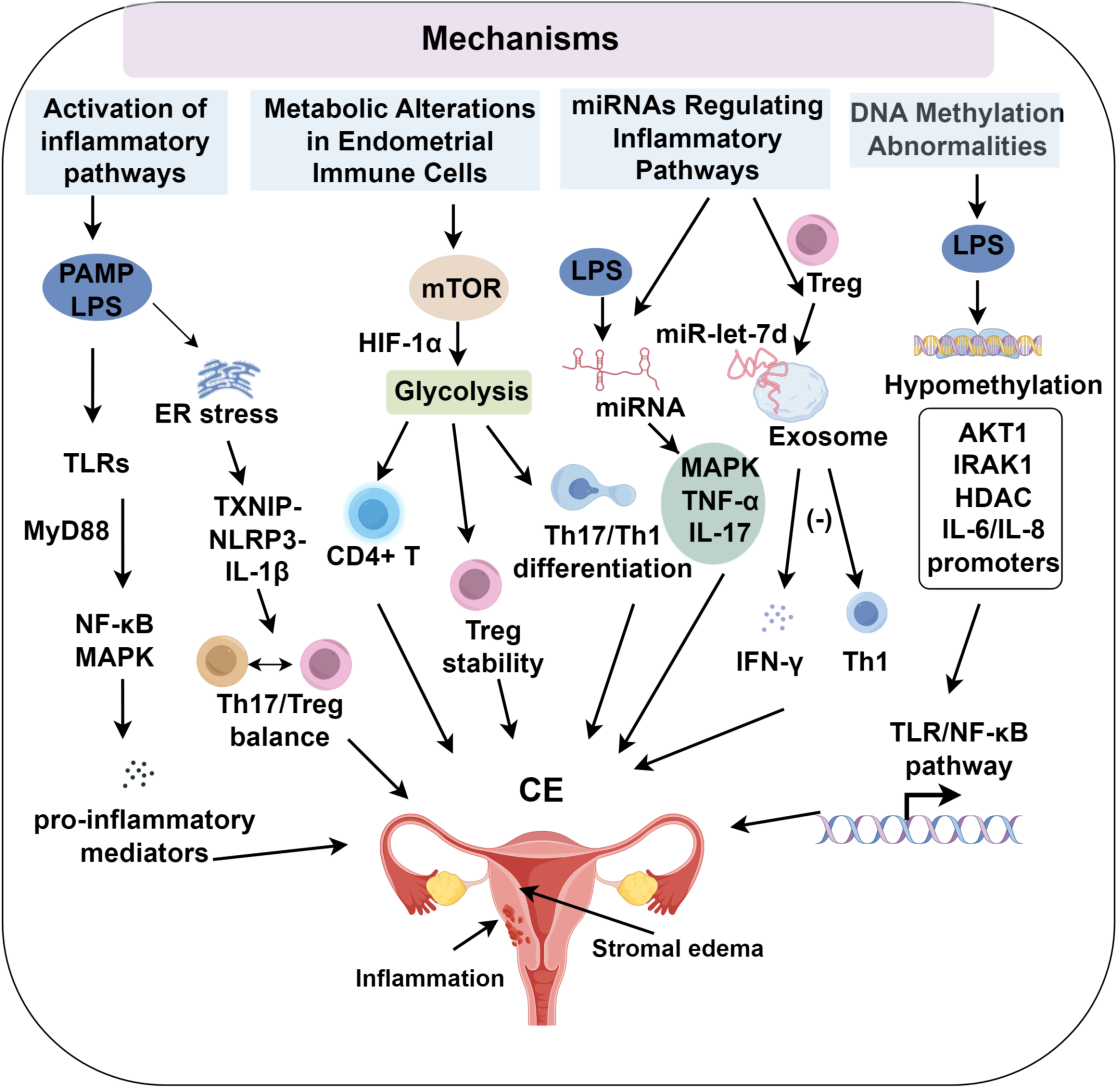
The role of inflammation in chronic endometritis progression.

miRNAs are small non-coding RNA molecules, typically 20 – 22 nucleotides in length, that primarily regulate gene expression post-transcriptionally by binding to the 3’ untranslated regions (UTRs) of target mRNAs, thereby inhibiting translation (61, 62). Research by Lv et al. (63) demonstrated that LPS stimulation of bovine endometrial stromal cells led to significant differential expression of miRNAs, which were notably enriched in the MAPK, TNF-α, and IL-17 signaling pathways. This suggests that miRNAs contribute to the inflammatory pathogenesis induced by LPS. miRNAs may influence the development of CE by modulating key molecules in these inflammatory pathways, specifically targeting transcripts such as IRAK1, TRAF6, and components of the MAPK and NF-κB pathways. This modulation leads to quantifiable changes in downstream cytokine expression levels and immune cell subset activation, including CD4^+^ Th1 bias or M1 macrophage polarization (27, 63–65). The regulation of miRNA and mRNA forms a complex network, with much of the current research on miRNAs in CE being conducted at the level of individual cell types (66). However, future studies are necessary to validate the role of miRNAs in CE, particularly through the use of uterine organoids or human endometrial tissues. Collectively, miRNAs represent an epigenetic interface that modulates canonical signaling networks, providing novel targets for diagnostic and therapeutic interventions in CE (**Figure 1**).

#### 3.3.2 Exosome-derived miRNAs modulating endometrial inflammation

Exosomes are extracellular vesicles secreted by host cells, including epithelial cells, stromal cells, and immune cells, as well as by microbes (67, 68). These vesicles carry proteins, lipids, mRNAs, and miRNAs, facilitating intercellular communication by transferring these molecules to target cells and modulating their functions (69). For instance, Treg cells release exosomes that transfer exosome-derived miR-let-7d to Th1 cells, inhibiting cell proliferation and γ-interferon secretion, thereby suppressing inflammation (70). Exosome-derived miRNAs in the uterine cavity fluid play a significant role in regulating inflammation in CE (61). Exosome-derived miRNAs in cattle with endometritis undergo dysregulation. For example, the secretion of miR-218 by BEEC exosomes decreases, reducing its inhibitory effect on MIP-1 expression in target cells, thus promoting inflammation. Furthermore, exosomes can exert substantial immune-modulatory effects by transferring PAMPs and other antigenic substances, contributing to the regulation of inflammation (27). Therefore, exosomes serve as important mediators of cell-to-cell communication, playing pivotal roles in immune dysregulation and the inflammatory response in CE. Exosome-derived miRNAs, as potent intercellular messengers, reinforce the inflammatory milieu in CE by linking intracellular regulation with extracellular communication.

### 3.4 DNA methylation abnormalities

Microbial infections can induce host cell DNA demethylation. LPS alters DNA methylation in BEECs, primarily causing hypomethylation and upregulation of protein-coding genes involved in immune function, inflammation, proliferation, apoptosis, adhesion, and extracellular matrix remodeling (71). These changes, including hypomethylation of AKT1 and IRAK1, activate the TLR/NF-κB pathway, contributing to LPS-induced endometrial inflammation (71). Moreover, LPS demethylates the promoters of IL-6 and IL-8, thereby enhancing their expression. Hypomethylation of HDAC genes leads to the upregulation of HDACs, exacerbating inflammation through modulation of lymphocyte signaling, stabilization of HIF-1α, and acetylation of TLR pathway molecules (72). Persistent infection-induced methylation changes in regulatory regions may drive chronic endometrial inflammation in patients with CE. The role of DNA methylation in the pathogenesis of CE remains unclear, although studies have shown menstrual cycle-dependent methylation dynamics in healthy endometria, with distinct patterns observed in endometriosis and carcinoma (73). Epigenetic reprogramming *via* DNA methylation acts as a persistent memory of inflammation, and its integration with miRNA and immune pathway data could provide valuable insights into the chronic progression of CE (**Table 1**).

**Table 1:** Inflammatory mechanisms in chronic endometritis.

Mechanism	Description	Pathways Involved	Molecular Targets	Implications for CE Pathogenesis
Microbial Dysbiosis	Imbalance in the endometrial microbiota due to pathogen colonization	TLR, NLR, NF-κB pathways	Streptococcus, E. coli, Ureaplasma, Enterococcus faecalis	Disrupts endometrial receptivity and immune regulation
Inflammatory Pathway Activation	Pathogen-associated molecular patterns (PAMPs) like LPS trigger immune responses leading to inflammation	TLR4/NF-κB, NLRP3 inflammasome	IL-1β, IL-6, TNF-α, Chemokines	Leads to immune dysregulation and chronic inflammation in the endometrium
TLR Pathway Dysregulation	Overactivation of TLRs results in an exacerbated inflammatory response	MyD88-dependent NF-κB, MAPK	TLR4, TLR2, NF-κB	Persistent inflammation in CE, exacerbating immune cell infiltration
NLRP3 Inflammasome Activation	NLRP3 inflammasomes activated by LPS-induced ER stress, leading to IL-1β production	NLRP3, IL-1β, Caspase-1	NLRP3, IL-1β	Contributes to chronic inflammation and immune cell activation in CE
Metabolic Reprogramming in Immune Cells	Shift towards glycolysis in T cells and macrophages that enhances inflammation	mTOR, HIF-1α, glycolytic enzymes	HIF-1α, PFK, mTOR	Accelerates immune cell activation, T-cell proliferation, and M1 macrophage differentiation
miRNA-Mediated Inflammation	Dysregulated miRNAs modulate inflammatory pathways and immune responses	MAPK, IL-17, TNF-α signaling	miR-146a, miR-155, miR-21	Drives inflammation by altering key inflammatory mediator levels
DNA Methylation Abnormalities	Epigenetic changes induced by microbial infections result in altered gene expression patterns	DNA demethylation of immune-related genes	AKT1, IRAK1, HDACs, IL-6, IL-8	Sustains inflammatory responses and immune cell dysfunction in CE

## 4 Treatment of chronic endometritis

### 4.1 Oral antibiotic eradication therapy

Management of CE has proven effective in normalizing endometrial histopathological features and improving reproductive outcomes in affected patients (74, 75). Current therapeutic strategies for CE involve three main approaches: empirical systemic antibiotic administration, intrauterine antimicrobial instillation, and probiotic supplementation to restore microbial balance (76, 77). Among these, oral antibiotic regimens remain the most widely used clinical approach. Cicinelli et al. (78) conducted a thorough evaluation of antibiotic protocols tailored for CE individuals with RIF. Johnston-MacAnanny et al. (79) reported that monotherapy with oral doxycycline resulted in clinical resolution in approximately 70% of RIF individuals with confirmed CE. In cases with doxycycline resistance, combination therapy using ciprofloxacin and metronidazole was effective in eliminating plasma cell infiltration from the endometrial stroma, as confirmed by histopathological examination of endometrial biopsies (80). However, despite appropriate antibiotic treatment, RIF individuals with CE consistently showed lower embryo implantation rates compared to non-CE counterparts. Current clinical guidelines, as outlined in the 2021 Sexually Transmitted Infections Treatment Guidelines, recommend an antibiotic regimen initially developed for pelvic inflammatory disease, including endometritis, which combines doxycycline with metronidazole (81).

### 4.2 Intrauterine infusion therapy

Intrauterine infusion represents a targeted therapeutic approach that enables direct medication delivery into the uterine cavity, overcoming the limitations of prolonged oral antibiotic regimens (77, 82). This localized delivery system offers several clinical advantages, including enhanced drug concentration at the target site, reduced systemic exposure, and improved cost-effectiveness (83). A clinical study assessing the efficacy of intrauterine antibiotic infusion combined with dexamethasone showed promising reproductive outcomes (84, 85). Comparative analysis revealed superior therapeutic results in patients with CE treated with intrauterine antibiotics compared to those receiving conventional oral combination antibiotic therapy (77). These findings suggest that the combined use of intrauterine antibiotics and corticosteroids constitutes an effective strategy for CE management, leading to improved pregnancy rates (85).

Beyond conventional antibiotic therapies, emerging evidence supports the use of intrauterine platelet-rich plasma (PRP) infusion as an effective treatment for CE (86). PRP, an autologous biological preparation containing concentrated platelets and bioactive molecules such as VEGF, PDGF, and TGF-β, exerts multiple therapeutic effects, including endometrial regeneration, anti-inflammatory action, and promotion of angiogenesis (87, 88). Clinical observations have demonstrated that PRP modulates the uterine immune environment by reducing endometrial populations of CD8^+^ T cells, CD56^+^ NK cells, Foxp3^+^ Treg cells, and T-bet^+^ Th1 cells in refractory CE cases (86). This immunomodulatory reprogramming correlates with enhanced endometrial receptivity and improved reproductive outcomes, even in antibiotic-resistant cases (86, 89). Notably, successful pregnancies have been reported following PRP treatment after failed antibiotic therapy (90), highlighting its potential as a salvage treatment.

## 5 Conclusion

CE is a multifactorial condition driven by a complex interplay of microbial infections, immune dysregulation, and epigenetic modifications, all contributing to impaired endometrial receptivity and adverse reproductive outcomes. The pathogenic mechanisms encompass pathogen-induced activation of TLR and NLR signaling pathways, metabolic reprogramming of endometrial immune cells, miRNA-mediated amplification of inflammatory responses, and aberrant DNA methylation patterns that sustain chronic inflammation.

Despite notable therapeutic advances, particularly the use of broad-spectrum antibiotics, persistent CE, treatment resistance, and recurrent reproductive failure continue to pose significant clinical challenges. Alternative approaches such as intrauterine infusion therapies, immunomodulatory strategies, and microbiome-based interventions have shown promising preliminary results. However, no unified consensus exists on treatment protocols, especially regarding the optimal antibiotic regimens, criteria for selecting intrauterine therapies, or the clinical application of emerging interventions like PRP. This lack of standardization contributes to considerable variability in treatment responses, limiting the comparability of outcomes across studies and complicating clinical decision-making. Future efforts must focus on addressing these gaps by establishing standardized diagnostic criteria and conducting multicenter randomized trials to evaluate combinatorial therapies and refine clinical management.

A deeper understanding of endometrial microbiota-immune interactions may facilitate the development of personalized therapies, improving pregnancy outcomes in patients with CE. Additionally, standardized diagnostic protocols and well-designed randomized trials assessing combination treatments are critical to optimizing clinical management and improving reproductive success. Exploring the causal relationships between specific microbial species and immune dysfunction through integrative multi-omics approaches could provide valuable mechanistic insights and support the development of targeted therapies.
